# Visuospatial working memory dysfunction from tapping span test as a diagnostic tool for patients with mild posterior cortical atrophy

**DOI:** 10.1038/s41598-021-90159-w

**Published:** 2021-05-19

**Authors:** Michitaka Funayama, Taketo Takata, Yoshitaka Nakagawa, Kosaku Sunagawa, Asuka Nakajima, Hiroaki Kawashima, Masaru Mimura

**Affiliations:** 1 Department of Neuropsychiatry, Ashikaga Red Cross Hospital, 284-1, Yobe, Ashikaga-City, Tochigi 3260843 Japan; 2 Department of Rehabilitation, Edogawa Hospital, Tokyo, 1330052 Japan; 3 Faculty of Rehabilitation, Kansai Medical University, Osaka, 5820026 Japan; 4 Department of Rehabilitation, Ashikaga Red Cross Hospital, Tochigi, 3260843 Japan; 5 Department of Neuropsychiatry, Keio University School of Medicine, Tokyo, 1608582 Japan

**Keywords:** Neuroscience, Neurology

## Abstract

Posterior cortical atrophy is a rare degenerative condition with prominent visuospatial dysfunction which commonly occurs between ages 50 and 65. A diagnosis of mild posterior cortical atrophy sometimes challenging and can be delayed because there are currently no established neuropsychological examination methods that can easily be used in clinical settings. In this study, we examined whether the tapping span test is a potential diagnostic tool for posterior cortical atrophy and what impairment the tapping span test is indicative of in this condition. Eight patients with mild posterior cortical atrophy were recruited. Age- and severity-matched individuals with amnesic Alzheimer’s disease (n = 9) were also recruited as a control group. The participants were subjected to the tapping span test and several visuospatial working memory tests. The results of the tapping span and visuospatial working memory tests were worse for the posterior cortical atrophy group when compared with the control group. The results from the tapping span tests were strongly correlated with those from the visuospatial working memory tests. The tapping span test is a simple and potentially useful diagnostic tool for patients with mild posterior cortical atrophy, as it reflects visuospatial working memory function.

## Introduction

Posterior cortical atrophy is a rare degenerative condition which commonly occurs between ages 50 and 65 and is characterized mainly by visuospatial dysfunction^1,2^. This condition is under-recognized and underdiagnosed^1–3^ because a diagnosis of posterior cortical atrophy is sometimes challenging among assessments of neurodegenerative conditions^3^, in particular, of the mild form and can be delayed not only because of the rarity of this condition but also because, aside from visuospatial dysfunction, patients with mild posterior cortical atrophy function almost normally with respect to memory, language, and behavior and maintain a relatively preserved motivation and insight^3–7^. In addition, typical simple neuropsychological tests, e.g., Mini-Mental State Examination (MMSE)^8^, do not focus on symptoms of this condition; rather, their goal is mainly to assess orientation, calculation, repetition, and memory functions. Although visuospatial function might in part be evaluated with a subtest in the MMSE, namely copying the intersecting pentagons, its score results in only 1 point of the 30-point scale associated with the MMSE. Thus, even if patients with posterior cortical atrophy receive regular neuropsychological examination, there is the possibility that their condition might not be diagnosed.

Visuospatial dysfunction is the most common feature for mild posterior cortical atrophy^2^. Based on a neuropsychological examination for visuospatial dysfunction, visuospatial working memory was found to be impaired for patients with posterior cortical atrophy when compared with those with amnesic Alzheimer’s disease^9–11^. Although most patients with posterior cortical atrophy have the neuropathology of Alzheimer’s disease as in amnesic Alzheimer’s disease, it is of importance to discriminate posterior cortical atrophy from amnesic Alzheimer’s disease because symptoms of posterior cortical atrophy differ substantially from those of amnesic Alzheimer’s disease and how to help and support patients with posterior cortical atrophy should be specifically designed for their difficulties^3^. Regarding working memory, several recent reports on degenerative disorders have focused on working memory function^12–14^, in which working memory decline was identified in those disorders even in patients with mild cognitive impairment^14^. According to Kirova et al.^14^, difference in working memory function can distinguish the three stages, namely, normal aging, mild cognitive impairment, and Alzheimer’s disease. In addition, a hallmark of logopenic variant primary progressive aphasia, another clinical form of a neurodegenerative condition with mainly Alzheimer’s disease neuropathology, is an impaired phonological working memory which is represented by repetition deficits^15^. Thus, assessments of various types of working memory in neurodegenerative conditions might be indicative of a specific impairment for each neurodegenerative condition. However, application of measuring visuospatial function in clinical settings is limited because of the complexity of these methods, for which computers are usually used.

In this report, we examined whether the tapping span test^16,17^—a simple neuropsychological test that has been widely used in clinical settings and takes only a few minutes to complete without computers—is a potential diagnostic indicator of mild posterior cortical atrophy. We also analyzed whether the results from the tapping span test reflect visuospatial working memory function for patients with posterior cortical atrophy.

## Methods

### Participants

Ethical aspects of this study were reviewed and approved by the Hospital Human Research Ethics Committee. This study was performed after obtaining informed consent from all participants and was performed in accordance with the Declaration of Helsinki. The study participants were recruited from the Cognitive Dysfunction Clinics associated with Ashikaga Red Cross Hospital and Edogawa Hospital, during the period from June 2015 to Nov 2020. Patients with mild posterior cortical atrophy (0.5 Clinical dementia rating^18^) were included. Patients with > 1 on clinical dementia rating were excluded because the aim of this study was to examine whether the tapping span test is a potential diagnostic tool for mild posterior cortical atrophy. Among 13 patients who met both the clinical and imaging-supported diagnostic criteria for posterior cortical atrophy^2^, eight individuals who were in the mild form, that is, with a clinical dementia rating of 0.5^18^, were recruited. Time period from onset of first symptoms to inspection day was 2.8 ± 1.7 years (range: 0 to 5 years). This time period is similar to that of patients with a clinical dementia rating of 0.5 on Nourhashémi’s report (2008), which was 2.8 ± 1.9 years^19^. Members of a control group were also recruited, that is, age- and severity-matched individuals with amnesic Alzheimer’s disease (n = 9) from the same clinics, all of whom developed their symptoms at < 70 years of age to be matched in age for those with posterior cortical atrophy and also received their first medical examination while their severity were still mild (0.5 clinical dementia rating). Time period from onset of first symptoms to inspection day of this group was 2.7 ± 1.2 years (range: 1 to 5 years). All patients were evaluated by neuropsychiatrists (MF and TT), each of whom had more than 15 years of experience in neuropsychology and clinical practice for degenerative disorders at the time of the study. Regarding the initial symptoms, seven patients had visuospatial dysfunction as their initial symptoms, e.g., difficulties with connecting cables, following lines of text while reading, rearranging equipment, organizing a room, or causing a traffic accident, and the remaining one patient had agraphia. At the time of the neuropsychological assessments, none of the eight individuals had paresis, sensory disturbance, homonymous hemianopsia, or cortical blindness. Regarding clinical diagnosis for the eight individuals with posterior cortical atrophy (PCA), six patients had early-onset Alzheimer’s disease, one patient had PCA-CBS (corticobasal syndrome), and the remaining one had PCA-prion. Molecular evidence was available for two patients: one patient with PCA-AD whose amyloid beta 1–42 level and phosphorylated tau level in her cerebrospinal fluid were consistent with the diagnosis of Alzheimer’s disease and the other with PCA-prion who had 14–3-3 protein in her cerebrospinal fluid. The other six patients with posterior cortical atrophy were followed up for a minimum of 2 years after the inspection (average years: 3.2 ± 1.2), which allowed us to clinically differentiate between different subtypes of dementia.

### Demographics and basic neuropsychological assessments

The demographic factors investigated were age, gender, level of education, and the number of years post-onset. Basic cognitive function was evaluated using the Japanese version of the MMSE ^20,21^ and the Hasegawa Dementia Rating Scale-Revised (HDS-R)^22^. The HDS-R is most frequently used to evaluate the basic cognitive function of patients with dementia in Japan and, similar to the MMSE, includes items that assess orientation, memory, repetition, and calculation as well as backward digit span and category fluency. The maximum number of possible points attainable for the HDS-R is 30, which is also the case for the MMSE. However, there are some differences between the two tests. The HDS-R puts more emphasis on memory function than the MMSE: 11 of 30 points account for memory function in the HDS-R, whereas 3 of 30 points account for this in the MMSE. The passing cut-off point for the HDS-R is 20/21, whereas that for the MMSE is 23/24^20–22^.

Visuospatial function was evaluated using the Japanese version of the Trail Making Test A (TMT-A)^23–25^. In the TMT-A test, participants are instructed to connect a set of 25 numbers written on paper in numerical order by a drawn line. Performance on this test was expected to be severely impaired for patients with posterior cortical atrophy owing to their visuospatial dysfunction^23^. The time limit for the TMT-A was set at 300 s. If the subject had not completed the test in this time, the trial was terminated, and a value of 300 s was used for statistical analysis. For patients with posterior cortical atrophy, visuospatial and linguistic functions were compared using perceptual organization and verbal comprehension scores in the Japanese version of the Wechsler Adult Intelligence Scale—third edition, which reflect visuospatial function and linguistic function, respectively^26,27^. The lowest computable score for the perceptual organization was 50. If the subject’s scale had not reached this score, a value of 50 was used for statistical analysis.

### Tapping span test

The Tapping Span subtest from the Clinical Assessment for Attention^28^ was administered. The Clinical Assessment for Attention is a validated, reliable, and standardized test that has been widely used to assess attention function in Japan for more than a decade. The configuration of the Tapping Span subtest is the same as that of Spatial Span Tapping Test in the Wechsler Memory Scale^12^ with a slight difference in positions of probes on the cardboard. This Tapping Span subtest consists of a piece of cardboard with nine spatially distributed probes, each consisting of a square that is 2 cm wide. Two conditions were included in this subtest, i.e., Tapping Span Forward and Tapping Span Backward. The examiner taps sequences of probes of increasing length that have to be repeated in the same (forward) or reverse (backward) order by tapping them. Both conditions have two trials for each length of tapping sequence. If a participant is able to repeat either of the two trials for a tapping sequence of a certain length, he or she is regarded as having passed for that length, e.g., a tapping sequence of three probes. These methods for tapping span tests are also used in Corsi Block-Tapping Task^29^. Regarding method of scoring, the maximum length of the tapping sequence that an individual can repeat is presented as the result of the tapping span test, which differs from that of Spatial Span Tapping Test in Wechsler Memory Scale^16^, the result of which is the number of total correct answers in the individual span test. The reason why we employed the Tapping Span subtest from the Clinical Assessment for Attention is that it is simpler and less complicated in clinical settings to use the maximum length of the tapping sequence than the number of total correct answers for the result of the tapping span test.

### Digit span test

To assess phonological working memory along with visuospatial working memory, the Digit Span Forward and Digit Span Backward tests from the Clinical Assessment for Attention^28^ were evaluated, the methods of which were quite similar to those used in the Wechsler Memory Scale^16^. In the forward condition, sequences of digits of increasing length have to be repeated verbally in the same order in which they were previously read aloud by the examiner. In the backward condition, the digit sequences have to be repeated in reverse order. In this task, both conditions have two trials for each length of digit sequences, e.g., 3–5–2 and 9–4–7 for sequences of three digits in the forward digit span test. If a participant is able to repeat either of the two trials for a digit sequence of a certain length, he or she is regarded as having passed for that length. The maximum length of a digit sequence that the individual repeats is considered the result of the digit span test. This scoring method is also different from that of Spatial Span Tapping Test in Wechsler Memory Scale^16^, the result of which is the number of total correct answers in the individual span test. The reason why we employed these subtests from the Clinical Assessment for Attention for digit span test is the same as the one for the tapping span test.

To compare the results of these two kinds of span tests, the subtraction of digit span from tapping span data was carried out for all participants.

### Visuospatial working memory test

The tapping span score has been considered to reflect visuospatial working function^29^. However, caution should be exercised when interpreting a tapping span score in terms of the visuospatial working memory when this test is applied to individuals with visuospatial dysfunction. An individual with severe visuospatial dysfunction is frequently considered to have Bálint syndrome, which includes psychic paralysis of gaze, optic ataxia, and dorsal simultanagnosia (an inability to perceive several items in a visual scene at the same time^30,31^. Optic ataxia, which indicates a difficulty in accurately reaching for an object under visual guidance despite having the normal limb strength required to do so, interferes with tapping span measurements because an individual with optic ataxia is unable to reach a probe with his or her hand even if they observe it visually. Considering the potential presence of optic ataxia, a test that requires no upper limb movement is preferable when assessing visuospatial working memory. In addition, psychic paralysis of gaze, an inability to voluntarily shift one's gaze to an object of interest despite unrestricted ocular movement, also negatively influences the results of a visuospatial working memory task. This occurs because an individual who has psychic paralysis of gaze is unable to visually perceive the probes in the tapping span test in the first place. Thus, to assess the relationship between the tapping span test and visuospatial working memory, a more elaborate paradigm is needed. To this end, a delayed visuospatial matching task and a shape-from-moving-dots task were used to assess visuospatial working memory^32^, neither of which involves upper-limb movement. Moreover, before we could investigate visuospatial working memory, it was necessary to assess how these individuals inputted visuospatial information. As is often the case with severe visuospatial dysfunction with psychic paralysis of gaze and/or severe dorsal simultanagnosia, individuals are sometimes unable to perceive even one or two objects at a time.

We therefore first assessed if the participants could perceive several objects at a time and correctly judge the positional relationships of those objects, that is, their very basic visuospatial function. Detailed explanations of these tasks are provided elsewhere^32^. In short, the judgment of positional relationships of two probes and three probes was used to assess the presence of either psychic paralysis of gaze and/or severe dorsal simultanagnosia. Each participant was asked to judge whether the position of a red probe was above or below that of a blue one for the condition with two probes and whether the position of a yellow probe was above, aligned with, or below with respect to the other two probes for the condition with three probes. There was a total of 30 trials for each of the two conditions.

Visuospatial working memory was then measured by two task types: a delayed visuospatial matching task for visuospatial short-term memory and a shape-from-moving-dots task for more active visuospatial working memory. Detailed descriptions of these tasks are provided elsewhere^32^. The delayed visuospatial matching tasks that we used were much simpler than those used previously, given the severe visuospatial dysfunction of patients with posterior cortical atrophy, and consisted of two levels, that is, visuospatial working memory for one location and two locations, both of which included 30 trials. After a study phase, participants were asked whether the locations of the probes were in the same place or not in the response phase. For example, for the condition with one location, a blue probe (a circle of 2.5 cm in diameter) was displayed for 2 s in the study phase. Then, the blue circle was removed and there was a 1-s delay; a new blue circle was then shown for the response phase, during which the participant was instructed to say “same” when the circles were in the same place and “different” when they were not. For the condition with two locations, a blue probe was presented for 2 s for the study phase, followed immediately by a second blue probe for 2 s. After presentation of the first and second probes, there was a 1-s delay, after which the participant was shown another blue probe (response phase) and the participant was then asked to state “same” if the location of the third probe was the same as one of the two probes or “different” if it was different from that of both probes. Probes were displayed on the vertical meridian on a computer screen, which circumvented the potential impact of unilateral neglect by the subjects. There was a total of 30 trials for each of the two conditions.

**Figure 1:**
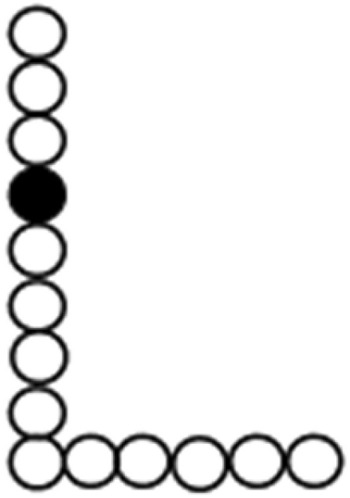
Visuospatial-temporal integration for shape-from-moving-dots. Each dot was presented for 1 s with no interval between presentations, i.e., the dots were displayed one at a time, which forced the subject to mentally integrate them across space and time to generate a complete object or letter, e.g., “L”.

In the shape-from-moving-dots task, participants were asked to name the shape generated by consecutively moving dots, which were displayed one at a time for 1 s each, which requires the participants to integrate them across space and time to generate a simple geometric object or a capital letter from the English alphabet (Fig. 1). This task included a total of 10 trials.

### Statistical analysis

The performance of individuals in the posterior cortical atrophy group were compared with those in the amnesic group. Excel 2010 with add-on Statcel 3 (OMS Ltd., Tokyo) was used for all statistical analyses. Significance was set at p < 0.05 (two tailed). Considering the small number of participants, variables were nonparametric and were compared between the two groups using the Mann–Whitney U-test**.** Gender distribution was compared between the two groups using Fisher's exact test. To investigate the relationship between the tapping span tests and visuospatial function/working memory tests and between the tapping span tests and digit span tests, Pearson’s correlation coefficient was used. For this analysis, data from the posterior cortical atrophy group and amnesic group were separately investigated because data from these two groups might be qualitatively distinctive.

### Patient consent for publication

This study was performed after obtaining informed consent from the patients.

### Ethics approval

Ethical aspects of this study were reviewed and approved by the Human Research Ethics Committee of Ashikaga Red Cross Hospital.

## Results

### Basic cognitive function

Table 1 shows the demographics and neuropsychological data for the posterior cortical atrophy group and the amnesic group of patients. Individual results of all 17 patients (8 patients with posterior cortical atrophy and 9 patients with amnesic Alzheimer’s disease) were presented in Tables 2 and 3. From clinical observation, no patients with posterior cortical atrophy had the complete form of Bálint syndrome and only had dorsal simultanagnosia while not having psychic paralysis of gaze or optic ataxia. Although two patients with posterior cortical atrophy declined to perform the Japanese version of the Wechsler Adult Intelligence Scale, the difference in performance between verbal comprehension and perceptual organization was substantial in six patients with posterior cortical atrophy (Mann–Whitney U-test, p < 0.01), which reflects their severe visuospatial dysfunction. No differences were found in the demographics between the two groups. Likewise, the results of the basic cognitive function and the digit span tests did not differ between the two groups, except for the TMT-A, which requires visuospatial function. Although there is no statistical difference in HDS-R between the two groups, the results of amnesic group was an average of 19.1 while those of posterior cortical atrophy was slightly higher at an average of 21.4, which might reflect memory dysfunction in patients with amnesic Alzheimer’s disease. Some patients gave relatively poor results on MMSE, which mainly reflected combination of mild cognitive decline and word-finding difficulties^33^ instead of moderate or severe cognitive decline.

**Table 1:** Demographics and neuropsychological data between posterior cortical atrophy and amnesic groups.

Attribute	Characteristic	Posterior cortical atrophy group (n = 8)	Amnesic group (n = 9)	P-value	Normal range for individuals in their 60 s
Demographics	Diagnosis	PCA(6), PCA-AD(1), PCA-prion (1)	Alzheimer (9)	–	–
	Age (year)	61.5 ± 5.6	61.4 ± 6.3	0.85	–
	Sex	Male 2, female 6	M 5, F 4	0.33	–
	Education (year)	13.3 ± 1.8	12.4 ± 1.3	0.24	–
Basic cognitive function	Mini-mental state examination	21.0 ± 3.5	21.7 ± 5.0	0.52	≥ 24 [21]
	Hasegawa dementia rating scale-revised	21.4 ± 4.9	19.3 ± 5.7	0.59	≥ 21 [22]
	WAIS verbal comprehension	85.5 ± 12.7	NA	NA	70-130 [27]
	WAIS perceptual organization	**58.3 ± 14.1**	NA	NA	70-130 [27]
	Trail making test [A] (s)	**265.0 ± 65.5**	174.4 ± 32.2	**0.01**	157.6 ± 65.8 [25]
Span tests	Tapping span forward	**2.1 ± 1.1** **(Range: 1 to 4)**	4.2 ± 0.7 (Range: 3 to 5)	**0.002**	5.6 ± 0.8 [28]
	Tapping Span Backward	**0.9 ± 1.2** **(Range: 0 to 3)**	3.1 ± 0.8 (Range: 2 to 4)	**0.003**	4.8 ± 1.5 [28]
	Digit span forward	5.6 ± 0.9 (Range: 4 to 7)	5.0 ± 1.0 (Range: 4 to 7)	0.16	5.8 ± 1.0 [28]
	Digit span backward	2.9 ± 0.6 (Range: 2 to 4)	3.1 ± 0.8 (Range: 2 to 4)	0.63	4.3 ± 0.9 [28]
Subtraction of digit span from tapping span	Forward	**3.5 ± 0.9** **(Range: 2 to 5)**	0.7 ± 1.1 (Range: − 1 to 1)	**0.001**	–
	Backward	**2.0 ± 1.1** **(Range: 0 to 3)**	− 0.1 ± 0.6 (Range: − 1 to 1)	**0.002**	–
Basic visuospatial function	Positional judgment of two probes	30.0 ± 0.0	30.0 ± 0.0	1.0	30.0 ± 0.0 [32]
	Positional judgment among three probes	24.6 ± 10.5	30.0 ± 0.0	0.12	30.0 ± 0.0 [32]
Visuospatial working memory	Delayed matching task in one-location condition	**19.9 ± 8.7**	28.9 ± 1.8	**0.011**	29.6 ± 0.9 [32]
	Delayed matching task in two-location condition	**13.1 ± 8.4**	25.3 ± 2.7	**0.0006**	25.7 ± 3.7 [32]
	Shape-from-moving-dots task	**1.3 ± 1.8**	5.4 ± 3.4	**0.01**	7.8 ± 1.9 [32]

The p-values in bold text are significant (i.e., < 0.05).

*AD* Alzheimer’s Disease, *NA* not applicable, *WAIS* Wechsler Adult Intelligence Scale.

Values in bold font represent those with statistical significance.

**Table 2:** Individual results of neuropsychological assessment for patients with posterior cortical atrophy.

Pt	Age	Sex	MMSE	TMT-A	WAIS		Tapping span		Digit span		Visuospatial working memory		
					VC	PO	Forward	Backward	Forward	Backward	Delayed matching task in one-location condition	Delayed matching task in two-location condition	Shape-from-moving-dots task
**1**	57	F	25	**300**	84	**50**	**1**	**0**	4	3	**15**	**17**	**1**
**2**	67	M	24	**300**	86	**< 50**	**2**	**0**	5	3	**26**	**14**	**0**
**3**	64	F	20	178	86	**66**	**3**	**2**	7	4	29	**20**	**2**
**4**	52	F	21	142	NA	NA	**1**	**0**	6	**2**	**15**	**0**	**0**
**5**	68	F	19	**300**	NA	NA	4	3	6	3	30	21	5
**6**	63	F	15	**300**	69	**< 50**	**1**	**0**	5	**2**	**5**	**0**	**0**
**7**	57	M	25	**300**	108	84	**3**	**2**	6	3	**24**	**18**	**2**
**8**	64	F	19	**300**	80	**< 50**	**2**	**0**	6	3	**15**	**15**	**0**
Avg	61.5		21	265.0	85.5	58.3	2.1	0.9	5.6	2.9	19.9	13.1	1.3
SD	5.6		3.5	65.5	12.7	14.1	1.1	1.2	0.9	0.6	8.7	8.4	1.8

Maximum time on the TMT-A was 300 s; participants not finishing with that time limit are indicated by 300.

*MMSE* Mini-Mental State Examination, *NA* not applicable, *TMT-A* Trail Making Test-A, *WAIS* Wechsler Adult Intelligence Scale, *VC* Verbal Comprehension, *PO* Perceptual Organization.

Values in bold font represent those of below 5% tile.

**Table 3:** Individual results of neuropsychological assessment for patients with amnesic Alzheimer’s disease.

Pt	Age	Sex	MMSE	TMT-A	Tapping span		Digit span		Visuospatial working memory		
					Forward	Backward	Forward	Backward	Delayed matching task in one-location condition	Delayed matching task in two-location condition	Shape-from-moving-dots task
**1**	63	M	24	178	5	3	5	3	30	27	6
**2**	57	F	25	135	5	4	5	3	29	27	9
**3**	64	M	24	213	5	4	6	4	30	28	9
**4**	52	M	25	165	**3**	2	4	3	30	23	**1**
**5**	61	F	13	150	4	2	4	**2**	**25**	22	**1**
**6**	70	F	24	217	4	3	5	3	30	21	8
**7**	56	M	13	137	4	4	4	3	**27**	27	**2**
8	71	F	22	211	4	3	5	3	29	25	5
9	59	M	25	162	4	3	7	3	30	28	8
Avg	61.4		21.7	174.4	4.2	3.1	5.0	3.1	28.9	25.3	5.4
SD	6.3		5.0	32.2	0.7	0.8	1.0	0.8	1.8	2.7	3.4

*MMSE* Mini-Mental State Examination, *TMT-A* Trail Making Test-A.

Values in bold font represent those of below 5% tile.

### Tapping span tests

The results of the tapping span tests for the posterior cortical atrophy group were significantly worse than those for the amnesic group, with lengths of only 2.1 ± 1.1 and 0.9 ± 1.2 probes attainable for Tapping Span Forward and Backward, respectively, in the posterior cortical atrophy group, which contrasted with those of 4.2 ± 0.7 and 3.1 ± 0.8 probes attainable in the amnesic Alzheimer’s group. This was also the case for one patient with posterior cortical atrophy who had agraphia instead of visuospatial dysfunction for his initial symptom; his tests resulted with a span of 2 for Tapping Span Forward and of 0 for Tapping Span Backward. The minimum length of the Tapping Span Forward was 1, which was observed in two patients with posterior cortical atrophy. This indicates that these patients did not have psychic paralysis of gaze or optic ataxia while having dorsal simultanagnosia because they were at least able to visually observe one circle 2 cm wide and to reach it accurately with their hands.

The difference between the Digit Span Forward and Tapping Span Forward for the posterior cortical atrophy group was substantial, with a span length of 3.5 ± 0.9, which contrasts with that of the amnesic group (0.7 ± 0.7). Basic visuospatial function measured by positional judgment tasks did not reach statistical difference. This is because the task was easy and induced ceiling effects. Still, two patients with posterior cortical atrophy were unable to judge the relative position of a probe among three probes, although they were able to judge its position relative to that of a second probe, that is, they were able to perceive a maximum of two items at the same time. This indicates that they had dorsal simultanagnosia, a key symptom of posterior cortical atrophy^34^, whereas they did not have psychic paralysis of gaze. All of the results of visuospatial working memory function were worse for the posterior cortical atrophy group when compared with the amnesic group. The fact that the posterior cortical atrophy group failed in the delayed matching task with the one-location condition even when those individuals were able to perceive at least two items at the same time indicates that their visuospatial working memory was impaired when compared with the very basic visuospatial function.

### Correlation between tapping span and spatial working memory tests

Tables 4 and 5 demonstrate the relationships between the tapping span tests and the visuospatial working memory tests and those between the tapping span tests and the digits span tests for each group, for which positive correlations were found between the tapping span tests and the visuospatial working memory tests. Regarding the posterior cortical atrophy group, strongly positive correlations were found between the tapping tests and the visuospatial working memory tests. Similarly, for the amnesic Alzheimer’s group, moderately positive correlations were found between the tapping tests and the visuospatial working memory tests. Conversely, no statistically significant correlations were found between the tapping tests and the digit span tests. Similarly, there were no statistically significant correlations between basic cognitive function tests (MMSE and HDS-R) and the tapping tests in either group (p > 0.05 for all of these comparisons; data not shown).

**Table 4:** Pearson’s correlation coefficient between tapping span and spatial working memory tests and between tapping span and digit span for the posterior cortical atrophy group.

	Tapping Span Forward	Tapping Span Backward	Delayed matching task in one-location	Delayed matching task in two-location	Shape-from-moving-dots task	Digit Span Forward	Digit Span Backward
Tapping span forward	1	**0.93**	**0.85**	**0.75**	**0.85**	0.61	0.62
Tapping span backward	0.93	1	**0.75**	0.64	**0.93**	0.58	0.51
Delayed matching task in one-location	**0.85**	**0.75**	1	**0.78**	0.68	0.49	**0.74**
Delayed matching task in two-location	**0.75**	0.64	**0.78**	1	0.65	0.21	**0.88**
Shape-from-moving-dots task	**0.85**	**0.93**	0.68	0.65	1	0.33	0.41
Digit span forward	0.61	0.58	0.49	0.21	0.33	1	0.40
Digit span backward	0.62	0.51	**0.74**	**0.88**	0.41	0.40	1

Bold values represent those with P-values of < 0.05.

**Table 5:** Pearson’s correlation coefficient between tapping span and spatial working memory tests and between tapping span and digit span for the amnesic Alzheimer’s group.

	Tapping Span Forward	Tapping Span Backward	Delayed matching task in one-location	Delayed matching task in two-location	Shape-from-moving-dots task	Digit Span Forward	Digit Span Backward
Tapping span forward	1	0.67	0.13	0.58	**0.68**	− 038	− 0.38
Tapping span backward	0.67	1	0.19	**0.69**	0.60	− 0.31	0.00
Delayed matching task in one-location	0.13	0.19	1	0.27	0.62	0.57	**0.71**
Delayed matching task in two-location	0.58	**0.69**	0.27	1	0.44	0.09	0.09
Shape-from-moving-dots task	**0.68**	0.60	0.62	0.44	1	0.19	0.00
Digit span forward	− 0.38	− 0.31	0.57	0.09	0.19	1	0.50
Digit span backward	− 0.38	0.00	**0.71**	0.09	0.00	0.50	1

Bold values represent those with P-values of < 0.05.

## Discussion

Our study demonstrated two findings. First, tapping span and visuospatial working memory were severely impaired among the individuals with mild posterior cortical atrophy when compared with the control group, and a strong relationship was found between tapping span and visuospatial working memory. Second, the difference between Digit Span Forward and Tapping Span Forward was remarkably larger in patients with posterior cortical atrophy when compared with the control group.

These findings indicate that tapping span tests may be a simple, useful diagnostic tool for patients with mild posterior cortical atrophy. Our study also found that patients with mild posterior cortical atrophy did not show psychic paralysis of gaze or optic ataxia, which, together with a strong relationship between tapping span and visuospatial working memory, indicates that the short length of the tapping span test in these patients mainly reflects visuospatial working memory dysfunction. Thus a tapping span test might be used as an indicator for posterior cortical atrophy, as is the short length of digits from digit span tests among individuals with logopenic variant primary progressive aphasia, which reflects the impaired phonological working memory observed in those patients^15^.

Both the tapping span and digit span tests have been used in clinical settings throughout the world^16^, and, thus, this method should be convenient for clinicians who specialize in neuropsychology. They would simply perform these two types of span subtests in the Wechsler memory scale for a patient with a degenerative disease to consider whether he or she has posterior cortical atrophy if he or she gives a poor result in the tapping subtest and/or there is a wide difference between the results of the digit span and tapping span subtests. In fact, the average span length among individuals with mild posterior cortical atrophy of only 2.1 for the Tapping Span Forward test was extremely poor, and the difference between the Digit Span Forward and Tapping Span Forward results was as high as 3.5 on average in our study. This large difference is similar to that of Trotta’s study^11^, in which the difference between the digit span forward and visuospatial cube span results among patients with posterior cortical atrophy was ~ 4, although their method for determining the visuospatial cube span used a visual presentation − verbal restitution method instead of the visual presentation − visual/finger restitution approach used in our study. In addition, they used cubes instead of the circles on a sheet that we used in our study. Our simple method might facilitate diagnosis of mild posterior cortical atrophy, which can be challenging^3–7^. Caution should, however, be taken as this method is just a task that might reinforce clinical characteristics, which surely carries more weight than a task for the diagnosis of posterior cortical atrophy. Still, we consider that the usefulness of the simple tapping span test in clinical settings is remarkable. In addition, visuospatial working memory dysfunction, which is easily evaluated with the tapping span test, might be a hallmark of posterior cortical atrophy as is phonological working memory dysfunction (verbal repetition deficits) in logopenic variant primary progressive aphasia, both of which constitute atypical presentation of Alzheimer’s disease^2,3,15^. These working memory dysfunctions we discussed in this article belong to short-term memories rather than those related to executive functions, which is associated with frontal cortex^35^ but not posterior cortex. Our findings might suggest that posterior cortex involves basic working memory functions, i.e., short-term memories, rather than executive functions which is disrupted by frontal cortex damage or degeneration^35,36^.

Our study has two main limitations that should be considered when interpreting the results. First, although visuospatial dysfunction is the hallmark of posterior cortical atrophy, other symptoms, e.g., visual agnosia^2,37^, agraphia^38^, alexia^39^, and acalculia^40,41^, might develop in these patients initially. For such individuals, the tapping span test score might not reflect their dysfunction and the role of this test might be limited. However, one patient with agraphia as his initial symptom was also impaired in the tapping span tests, suggesting that they may be useful at least for patients that present mainly with agraphia. There is, however, a possibility that patients with marked atrophy in temporo-occipital cortices, whose symptoms frequently consist of visual agnosia or prosopagnosia, might not present with visuospatial working memory dysfunction, and, therefore, they may have a good result on the tapping span test. Although these cases are not common^2^, this possibility limits the role of this test in using as a routine clinical practice Second, as we mentioned earlier, these tapping span tasks are not suitable for patients with optic ataxia and/or psychic paralysis of gaze, which might also limit the availability of these tasks. However, among the three features of Bálint syndrome, silmultanagnosia is thought to be an early finding of posterior cortical atrophy as in our study, but not optic ataxia or psychic paralysis of gaze^42^. Thus, we consider that the tapping span test is useful at least for patients with visuospatial dysfunction, which is the hallmark of posterior cortical atrophy. Third, our results were derived from a small number of patients, and a further study with a large number of patients will be needed to establish this method as an indicator to support the diagnosis of posterior cortical atrophy.

## Conclusions

The performance of individuals with mild posterior cortical atrophy on the tapping span test was remarkably poor when compared with that of the amnesic control group. The tapping span test may be a simple, useful tool for diagnosing patients with mild posterior cortical atrophy, as it reflects visuospatial working memory function.

## Data Availability

The datasets generated and/or analyzed during the study are available from the corresponding author (MF) upon request.
